# Supplementary material for: Mechanistic neutral models show that sampling biases drive the apparent explosion of early tetrapod diversity
Source: Nat Ecol Evol. 2023 Jul 27;7(9):1480–9. doi: 10.1038/s41559-023-02128-3 (PMC10482683; doi:10.1038/s41559-023-02128-3)
Supplement: Supplementary file 1 — Supplementary Figs. 1–5. [file 41559_2023_2128_MOESM1_ESM.pdf]

nature ecology & evolution

Article

https://doi.org/10.1038/s41559-023-02128-3

Mechanistic neutral models show that sampling biases drive the apparent explosion of early tetrapod diversity

In the format provided by the authors and unedited

Contents

Supplementary figure S1 2

Supplementary figure S2 3

Supplementary figure S3 4

Supplementary figure S4 5

Supplementary figure S5 6

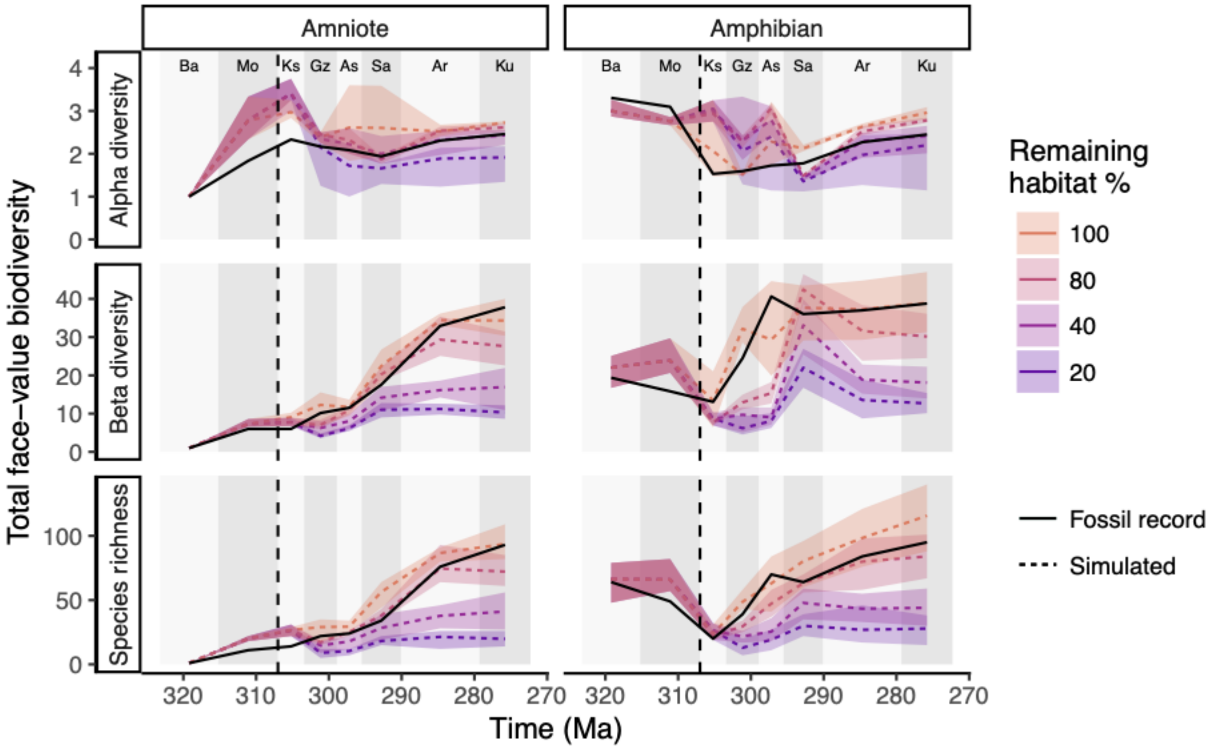

Figure S1: Simulated tetrapod diversity patterns over time compared against the fossil record (i.e. face-value, unstandardized counts of species). Predictions of tetrapod biodiversity patterns are produced by a neutral model parameterised with 80% habitat remaining (20% loss) and then simulated with different levels of remaining habitat (i.e. 100%, 40% and 20% habitat remaining). The shaded areas surrounding the dashed lines represent the variation in the five best fitting simulations. The dashed vertical line at 307 Ma indicates the timing of the CRC. For definitions of diversity measures see Table 1. The following abbreviations are used for intervals: "Ba" = Bashkirian, "Mo" = Moscovian, "Ks" = Kasimovian, "Gz" = Gzhelian, "As" = Asselian, "Sa" = Sakmarian, "Ar" = Artinskian and "Ku" = Kungurian.

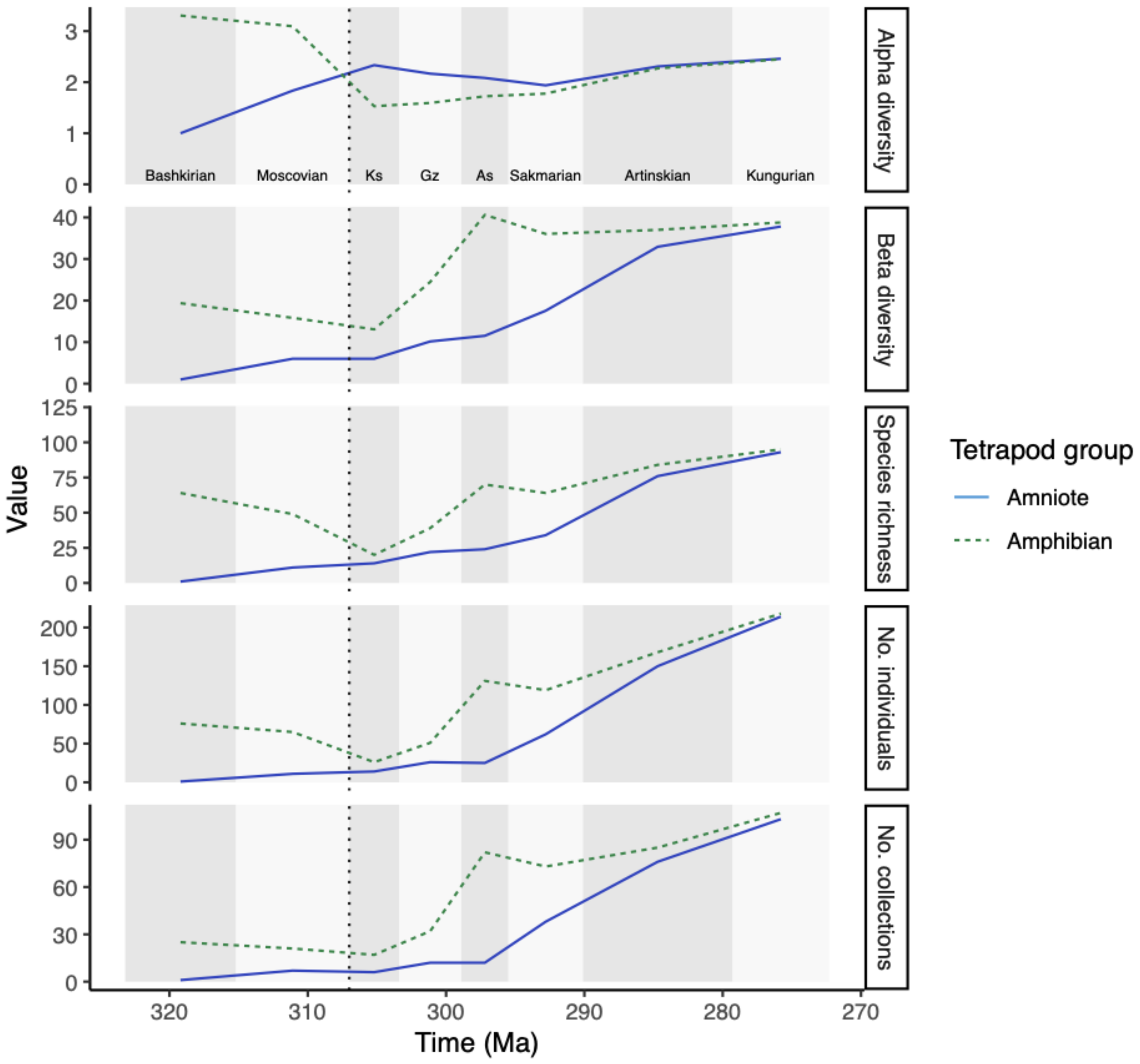

Figure S2: Raw data from the fossil record, indicating biodiversity metrics (alpha diversity, beta diversity and total species richness) and the number of individuals (i.e. fossils) and collections over time. Interval abbreviations are as in Figure S1.

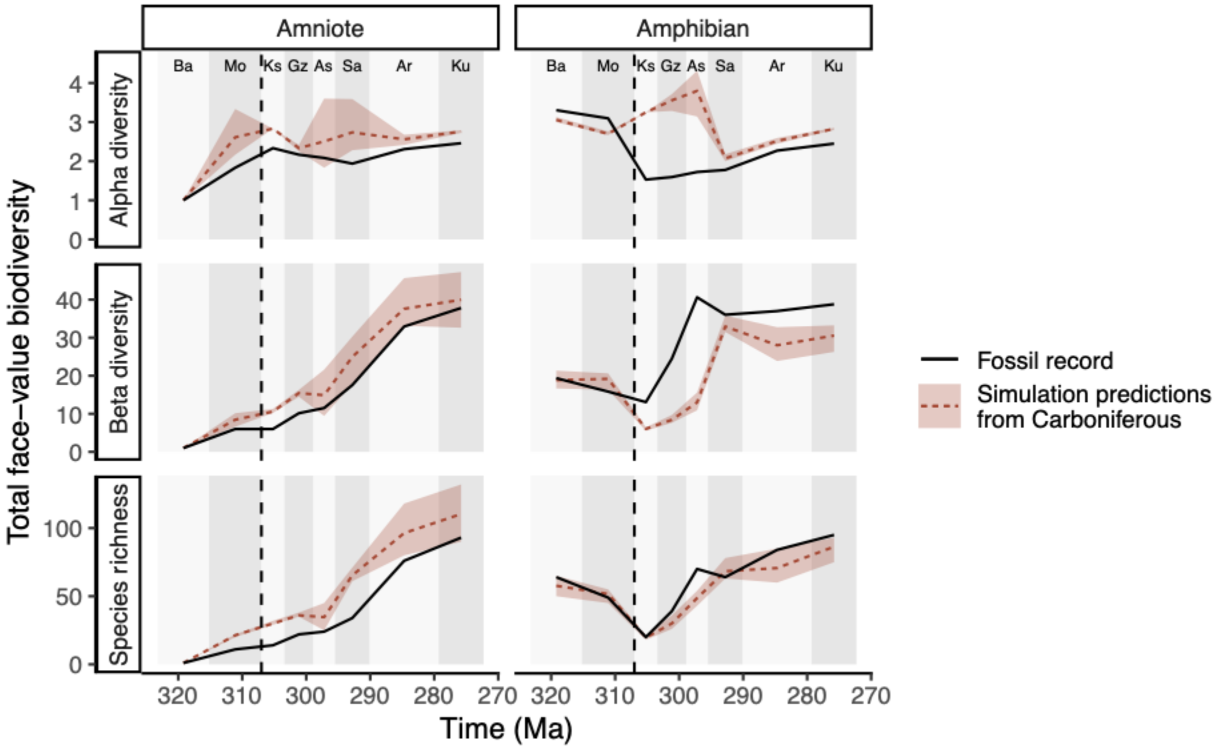

Figure S3: Simulated tetrapod diversity patterns over time compared against the fossil record (i.e. face-value, unstandardized counts of species). Predictions of tetrapod diversity from a neutral model parameterised solely on Carboniferous diversity. Three metrics of biodiversity (alpha, beta, and gamma diversity; Table 1) are shown for both amphibians and amniotes from the Bashkirian to Kungurian from empirical data (solid black lines) and from simulated communities (dashed lines). The shaded areas surrounding the dashed lines represent the variation in the five best fitting simulations. The dashed vertical line at 307 Ma indicates the timing of the CRC. For definitions of diversity measures see Table 1. Interval abbreviations are as in Figure S1.

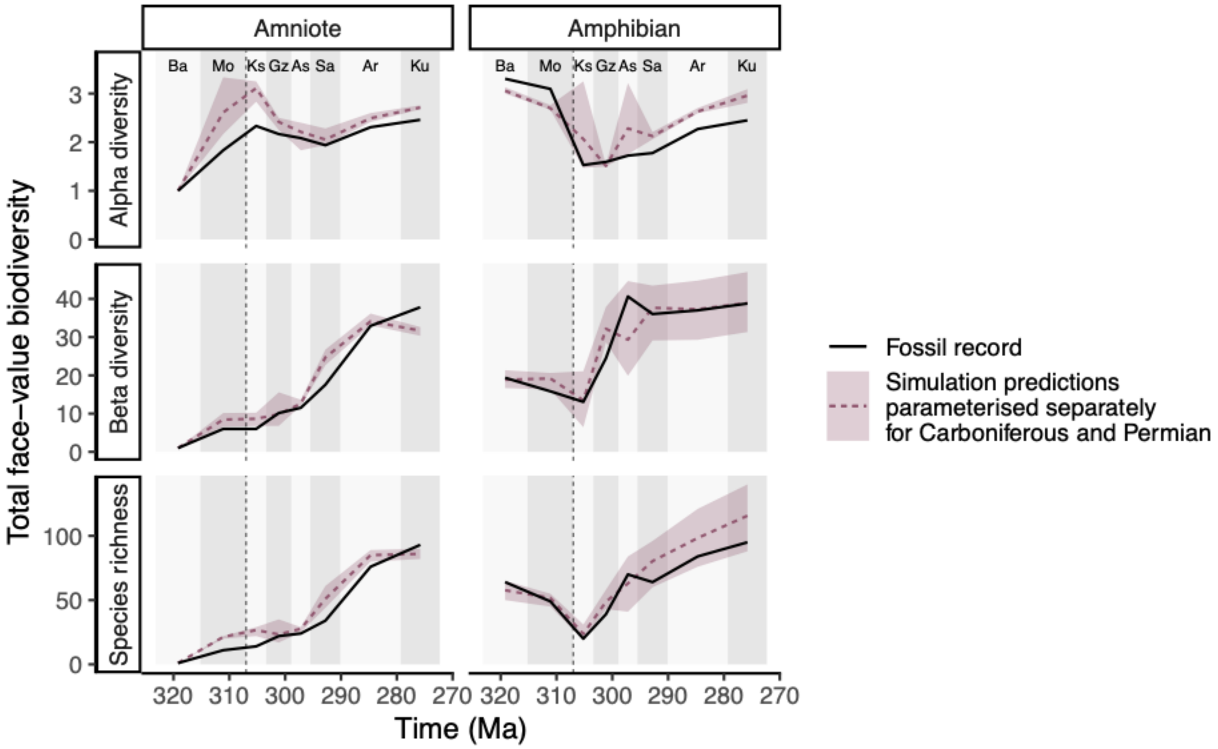

Figure S4: Simulated tetrapod diversity patterns over time compared against the fossil record (i.e. face-value, unstandardized counts of species). Predictions of tetrapod biodiversity patterns are produced by a neutral model parameterised separately for the late Carboniferous (pre-307 Ma) and Permian (post-307 Ma). The shaded areas surrounding the dashed lines represent the variation in the five best fitting simulations. The dashed vertical line at 307 Ma indicates the timing of the CRC. For definitions of diversity measures see Table 1. Interval abbreviations are as in Figure S1.

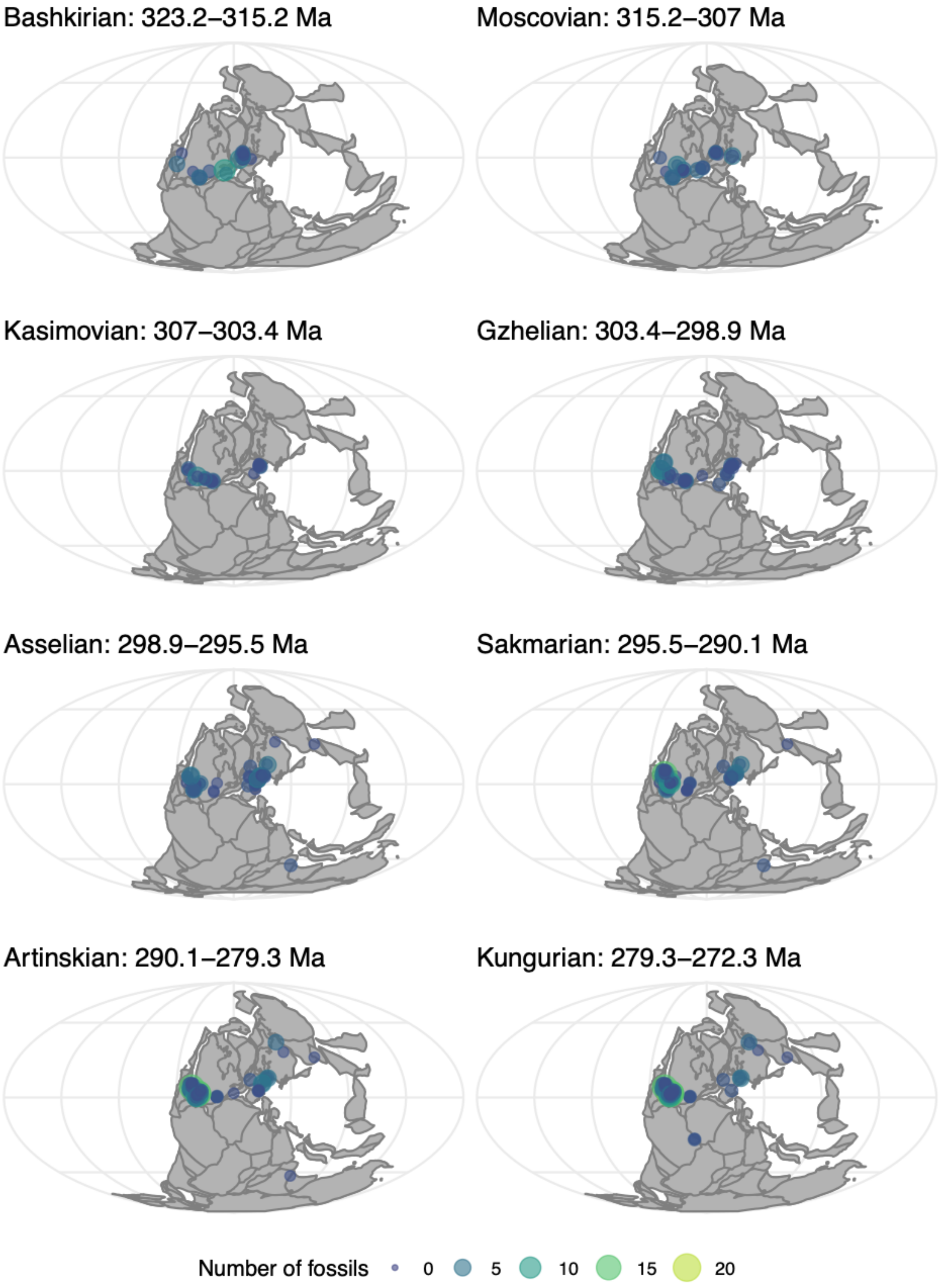

Figure S5: Global palaeogeographical maps showing the localities of fossil sites in each stage of the late Carboniferous and early Permian. The size and colour of each circle corresponds to the number of species found at each site. Continental configurations are provided by GPlates via the chronosphere R package.
